# New Nurses’ Experience of Caring for COVID-19 Patients in South Korea

**DOI:** 10.3390/ijerph18189471

**Published:** 2021-09-08

**Authors:** Eun-Joo Ji, Young-Hee Lee

**Affiliations:** Department of Nursing, Catholic Kwandong University, Gangneung 25601, Korea; 93eunjoo@cku.ac.kr

**Keywords:** COVID-19, nurse, communicable disease, qualitative research

## Abstract

The purpose of this study was to explore the experiences of new nurses who took care of COVID-19 patients. For this study, study subjects were conducted with a total of nine new nurses, and data were collected through individual in-depth interviews from September to November 2020. The data were analyzed using the phenomenological analysis method suggested by Colaizzi. New nurses’ experience of caring for COVID-19 patients consisted of three categories. The three categories are “The fear as a new nurse about infectious diseases that they have not experienced”, “Physical and psychological burden in an isolated environment”’, and “Building professional values”. Findings from this study presented vivid experiences of new nurses who took care of COVID-19 patients. This study is meaningful in that it grasped the physical and psychological difficulties of nurses nursing COVID-19 patients, especially the difficulties as a new nurse, and the implications for developing and growing within them. It is expected that it will serve as basic data for the establishment of strategies for infectious education programs for new nurses.

## 1. Introduction

Coronavirus disease 2019 (COVID-19) was first reported in Wuhan, China in late 2019. It became an unprecedented disease in the 21st century and as of 2021, there have been over 100 million confirmed cases and 2.35 million deaths worldwide, including 81,000 confirmed cases and 1,400 deaths in South Korea alone [1]. The virus began to spread quickly throughout South Korea from January 2020, as a result of which the country transitioned to a non face-to-face society to prevent the spread of infection, alongside changes in work and labor structure. Moreover, South Korea’s economy was severely affected, with the economic growth rate being −1% in 2020 [2]. With people experiencing an infectious disease that will leave a long-lasting mark on the history of civilization, time will now be divided into pre- and post-COVID-19 eras [3].

As of 2021, it is still difficult to predict when the pandemic will end. Even if this viral infection ends, there is a high likelihood of a new respiratory infection emerging at any time. While the medical community is expected to play an important role in such a situation, it is facing the difficult reality of shortage and burnout of nurses and other healthcare workers [4]. Twenty thousand new nurses graduate each year from nursing colleges in South Korea in accordance with government guidelines [5], but the number of active nurses per 1000 population is 3.8, which falls short of the Organization for Economic Co-operation and Development average of 7.6/1000 population [6]. Accordingly, the nursing community is facing greater difficulties due to COVID-19, due to which even more nursing personnel are needed.

Since SARS-CoV-2 can be transmitted via air, droplets, and contact under specific conditions, it is recommended that full-body personal protective equipment (PPE; level D) should be worn in medical institutions [7], which requires special education. However, because of the need for a large scale workforce, various problems have surfaced, including the dispatch of nurses being required to learn their role and perform their duties on their own without any education in advance [8].

Based on previous experience with infectious disease outbreaks such as H1N1 influenza in 2009, Middle East respiratory syndrome (MERS) in 2015, and COVID-19 in 2019, it is not difficult for humans to predict other emerging infectious disease outbreaks they will face in the future. The role of nursing education institutions that train nurses during such situations is highly important. However, aside from a general course on infection management, a systematic education on infectious diseases is not actually taking place, and even infection education provided at the nursing college level is limited [9]. Moreover, because students are not allowed to acquire experience with patients who present a risk of infection during their clinical practice training, most students have no experience with actual infected patients before graduating [10]. Since infection cases were concentrated in the medical environment during the MERS outbreak in 2015, the risk of infection was high among healthcare workers [11] and especially high among nurses with little hospital experience [12]. People in the field emphasized the need for education and training for proper response to emerging infectious diseases [13], which has also raised the need for the aforementioned systematic education.

New nurses are in a transition stage from nursing graduates to actual nurses. They not only lack the professional knowledge and skills required for nursing practice, but also face difficulties in applying theoretical knowledge to actual work performance [14]. Since the current clinical practice curriculum consists of observation-based education, measurement of vital signs, repetition of simple tasks, and patient transport [15], there is no opportunity to directly perform nursing techniques on actual patients, meaning that there is a high likelihood of performing most of the nursing techniques for the first time in the clinical setting where the nurses are assigned after graduation. Moreover, they experience stress due to difficulties in interpersonal relationships, burdens and anxiety about work, and lack of professional competency [16,17]. The present study conducted a phenomenological research method analysis for contextually understanding the experiences of nurses, focusing on their experiences of caring for COVID-19 patients in situations where they lacked appropriate knowledge, skills, and attitudes. 

While the majority of studies on those who participated in caring for patients with emerging infectious diseases in South Korea have examined nursing managers [18] and nurses [19,20], it is difficult to find studies on new nurses. Accordingly, the present study aimed to explore the experiences of the new nurses who took care of COVID-19 patients by identifying the meaning and essence of vivid patient care experiences of new nurses who were vulnerable and did not have much infectious disease experience.

## 2. Materials and Methods

### 2.1. Design

The present study used Colaizzi’s [21] phenomenological research method for an in-depth understanding of new nurses’ experience of caring for COVID-19 patients.

The phenomenological method is an inductive method for identifying the meaning and essential structure of subjective experience through statements about human experience in a certain situation, where the researcher accepts and understands the subjective perspective of the participants without any preconception or prejudice [22].

### 2.2. Participants

The participants consisted of new nurses engaged in three-shift work at general hospitals in Seoul, Andong, Daegu, and Gangneung, South Korea. Inclusion criteria were: (1) nurses with more than six months but less than two years of work experience after graduation and (2) those who had experienced caring for COVID-19 patients for at least two weeks during the COVID-19 pandemic. The study used snowball sampling, wherein a nurse who volunteered to participate in the study would introduce their nurse colleague(s). The study was explained to all candidates by the researcher via telephone and those who consented to participate in the study were subsequently interviewed. The final study sample (*n* = 9) had clinical experience ranging between 6 to 18 months.

The participants worked in designated COVID-19 hospitals or designated wards. All nine participants (five females and four males), age ranging from 24–27 years, were single and had an education level of a bachelor’s degree. With respect to work experience, three participants had less than one year of experience, while six participants had 1–2 years of experience.

### 2.3. Data Collection 

The data collection period was between September and November 2020. Since data collection took place during the time when social distancing was strictly enforced, individual interviews were conducted online and offline due to the risk of infection. Offline interviews were conducted in coffee shops or college research laboratories where the participants could talk comfortably at the preferred time and place without any concern of infection. Online interviews were conducted using a video conferencing program to view the faces and actions of the participants.

The interview followed an unstructured format. The main question was: “What did you experience when providing care after being assigned patients with emerging respiratory infectious disease or how did you feel?”. Participants were instructed to answer the question by recalling their first assigned care experience. Each interview lasted 60–90 min and the participants were given enough time to respond to the question. One to two interviews were conducted per participant and each interview was conducted until no new content could be extracted. The interviews were recorded with the consent of the participants. The recorded content was then inputted into a computer and transcribed. Subsequently, the transcript was read to ensure that it matched the interview content and was analyzed accordingly. All participants were given a small token of appreciation for their participation.

### 2.4. Ethical Considerations

The present study was approved by the Institutional Review Board of Catholic Kwandong University (NO-20-01-0107). After obtaining a signed informed consent form from each participant prior to data collection, one-on-one in-depth interviews were conducted. The participants were informed about their right to withdraw from the study at any time without any negative consequences, that their interviews would be used for research purposes only and would be well protected, and that all collected data would be destroyed after three years.

### 2.5. Data Analysis

The phenomenological research method by Colaizzi [21] was used on the collected data. In the first stage, the interviews were transcribed, wherein the researcher repeatedly listened to the transcribed content to understand the overall meaning of the participants’ experiences. In the second stage, meaningful statements within the transcribed content were underlined. As a result, 104 significant statements were derived from the nine participants. In the third stage, meaning was once again derived from the statements using the researcher’s own words, focusing on what the essence of caring for COVID-19 patients by new nurses was. In the fourth stage, meanings were derived from the themes. In the fifth stage, theme clusters were derived. In the sixth stage, themes and theme clusters were combined to form categories.

### 2.6. Rigor of Research

The present study ensured reliability and validity with respect to truth value, applicability, consistency, and neutrality, which are the elements of rigor proposed by Guba and Lincoln [23]. First, to increase the truth value, raw data were repeatedly read and verified to check whether the study results matched the interview content of the participants. Moreover, to reduce interinvestigator differences with respect to the data analysis results, an exchange of opinions between investigators took place and three participants checked the analysis results. Second, for applicability, data were collected until data saturation was reached. Moreover, the study results were presented to one nurse who also cared for COVID-19 patients—a fourth person who did not participate in the study—to check whether they could empathize with the results. Third, the consistency of data was maintained through a constant comparison method for analytical thinking in which components and sub-components identified in the data, study methods, data collection, and analytical processes were described in detail. Moreover, the researchers met regularly during the study period to share and exchange opinions on the characteristics of participants, approach methods, data collection method, and analytical methods. Fourth, to maintain neutrality, a conscious effort to not be affected by any prejudice was made by reviewing it through regular discussions among researchers to eliminate prejudice or bias due to the knowledge or experience of researchers. In particular, this was carried out by taking memos and putting parentheses on the prejudice that nurses who cared for COVID-19 would have a difficult time. Moreover, if the meaning of a participant during the interview was ambiguous, an additional question was added to clarify the meaning, while a decision trail was constructed to identify the influence of the researcher on the study process through communication among researchers.

## 3. Results

The study results of the experience of caring for COVID-19 patients by new nurses were summarized by fourteen themes, six theme clusters, and three categories (Table 1). The three categories that were identified consisted of the following: (1) “fear as a new nurse who has not experienced infectious disease,” (2) “physical and psychological burden in isolation environment,” and (3) “building professional values.”

### 3.1. Category 1: Fear as a New Nurse Who Has Not Experienced Infectious Disease

The participants did not experience caring for patients with infectious diseases during their clinical practical training and had no experience even after becoming a new nurse. In particular, the clinical progression of COVID-19, an emerging infectious disease, is not known. Accordingly, for nurses with less than two years of experience who must personally provide care, such inexperience itself became a difficulty. Moreover, the nurses felt afraid for people around them, including family members, becoming infected because of them.

#### 3.1.1. Difficulty from Being Inexperienced in New Clinical Duties

In their clinical practice training, the participants did not experience caring for patients with infectious diseases due to the risk of infection. Moreover, because of the unknown disease progression of COVID-19, they were fearful of how to provide care. They also stated that, as new nurses, they lacked knowledge. Consequently, they feared making decisions that required caring for patients with an infectious disease such as whether to first don protective equipment or check the patient in emergency situations.

“I don’t know about infectious diseases, and I encountered it for the first time, so what should I do…” (Participant #2)

“Honestly, I was scared and wanted to quit… I wasn’t sure what was going to happen.” (Participant #6)

“I really should study a lot… From the knowledge aspect, I need to prepare and learn a lot more.” (Participant #8)

“Will I be going to perform CPR if asystole appears on the patient monitor… It takes 5 min to don the protective equipment… Does that mean I can’t do CPR on the patient for 5 min or do I need to rush in there after simply putting on four basic protective equipment…” (Participant #1)

#### 3.1.2. Fear of Being a Carrier of Infection

The participants worried about whether the PPE they were wearing would protect them if respiratory secretion splashed on them. They also had a fear of becoming infected while working as a nurse, and then spreading the infection to others by being a carrier.

“A grandma coughed a lot and had a significant amount of phlegm discharge. All of that gets on your face shield… I was worried even though I was wearing PPE.” (Participant #4)

“Because I am a nurse, I am concerned about spreading the infection to people I have met today and the restaurants I went to today. If they become infected, it will have a devastating impact, so I’m concerned about those things.” (Participant #3)

“I keep my mask on when meeting my family members. I didn’t go to visit relatives such as my grandma, and grandpa. I rarely met with friends and lived pretty much by myself.” (Participant #1)

### 3.2. Category 2: Physical and Psychological Burden in Isolation Environment

The nurses had 6–18 months of experience and endured difficulties in performing IV injection or aspiration while wearing level D PPE, as they lacked the necessary skills. Due to their inexperience in using PPE, they became flustered when their PPE got damaged. As the COVID-19 pandemic continued, they were afraid that they may forget some of the nursing skills they had previously learned. Moreover, because a limited number of staff was to provide care in isolation wards, which could be entered only by those wearing PPE, they felt burdened by having to do other work, such as cleaning and other duties. While it was a difficult situation for all, being the youngest staff member made it even more difficult.

#### 3.2.1. Difficulties Due to Lack of Professional Skills

The participants lacked the skill to perform IV injections because of little clinical experience and found that having to perform them while wearing three layers of gloves and goggles that fog up was the most challenging part. They also felt the pressure of having to succeed, no matter what, while wearing the PPE. Leaving the room to remove the goggles that had fogged up during patient care and re-entering after donning new PPE or suddenly exiting the room when the batteries in the pressurized air circulation device went out were areas of major concern. They feared the possibility of forgetting the nursing skills that they had learned in school due to a lack of opportunities to use them since they were only applying the skills that were repeatedly needed under the COVID-19 situation.

“Since I was wearing two pairs of gloves, securing an IV line was very difficult… even more for older patients… It was difficult since it was not something I did a lot.” (Participant #5)

“I am wearing a mask and goggles, I was nervous, and it was a slightly frustrating situation… Normally, if I fail, I can ask someone else. But here, if I fail someone else has to come in wearing level D PPE. That burden made securing an IV line that much more difficult.” (Participant #6)

“Things like oxygen connectors run on batteries. When they run out, the alarm goes off because of it. I had to run down from the third floor to the first floor…” (Participant #4) 

“There are basic skills when you talk about a hospital with friends. I can’t do that since the COVID-19 outbreak. As a nurse, you should be able to do this and that. There is a slight sense that I’m being left behind. Since I’m doing only the COVID-19 work. I feel like I may be losing some of my nursing knowledge.” (Participant #6)

#### 3.2.2. Burden Due to Playing too Many Roles as a Nurse and the Youngest Member of the Ward

The participants felt overburdened by work because most duties were delegated to nurses since only a limited number of persons wearing PPE could enter the room. They faced a physical and psychological burden because of being the youngest member in the ward. 

“One more doctor going in increases the risk of infection, so only the nurses do the work. Controlling CRRT should be done by a cardiology nurse, but whichever nurse is in charge takes care of it. Things like cleaning that assistants or cleaning staff used to do were all done by nurses.” (Participant #1)

“A small number of nurses are going around, but many need to go in. Someone needs to keep an eye on the computer. I am way weary of others… Since I was working with experienced senior members, there was a sense of the youngest having to take care.” (Participant #3)

“We were doing a tracheotomy… It took over 2 hours. My lower back was really hurting, and I was drenched in sweat… But, when I came out, there was no chair for me to sit in. My lower back was hurting, but all the chairs were taken by the senior staff.” (Participant #1)

### 3.3. Category 3. Building Professional Values

The participants grew with respect to their knowledge, attitudes, and skills as they cared for COVID-19 patients. They were learning as they provided direct patient care under the given situation, with a sharp increase in COVID-19 patients. Moreover, they were building professional values, as they recognized their social role: performing important work within society.

#### 3.3.1. Growing and Learning with Respect to Knowledge, Skills, and Attitude

They attempted to use their knowledge to provide patient care by checking objective values, such as blood test results, even when the patients did not complain of any symptoms. They also attempted to combine their knowledge and clinical aspects to reduce unnecessary work. From a skills aspect, they repeatedly donned and doffed PPE for strict infection control. With respect to attitude, they reminded themselves to not have a biased view about the background of patients to ensure that they were providing equitable care to all. Nurses became aware of the fact that COVID-19 patients sometimes experience delirium while under isolation, and as a result, they recognized the need to provide not only physical care, but also emotional care, putting forth the effort to provide any necessary care.

“Because there are situations where the patient may look fine on the outside but suddenly change for the worse, I constantly monitored objective values and reported them to a doctor…” (Participant #7)

“Despite efforts to follow the guidelines, mistakes are always made in the field with regard to donning and doffing level D PPE… The subjects reported to infection control team members, who shared their thoughts on the problem regarding the donning and doffing processes despite repeated practice… Changes were also made accordingly.” (Participant #3)

“The same regular patient… Whether that person’s religion is Shincheonji or not, I should not be biased and provide care without discrimination as a patient who has the same disease.” (Participant #4)

“Isolated patients may experience delirium… Emotional care is more important than I thought… I realized it as I became a nurse… I paid close attention to things like that.” (Participant #7)

“There is no concept of time inside the hospital room, so I placed a calendar and a clock in the room. I also made them a fan since they said it was too hot.” (Participant #1)

#### 3.3.2. Recognized the Social Role of Performing Important Work

The participants recognized that the work they performed represented important work during the pandemic and that the general public had a positive perception about nurses. Through this, they also recognized their own social role. Conversely, they recognized the problem of inadequate compensation obscured by a sense of duty and stated the need for measures to address this issue.

“When I saw the ‘Thanks to You Challenge’ poster hanging on a bus… I felt proud in knowing that I’m doing meaningful work for the society.” (Participant #9)

“Because of the current COVID-19 pandemic, the country needs essential workers, and I am one of those workers. I believe it is an opportunity to develop my professionalism.” (Participant #7)

“I thought that the compensation does not match the sense of duty I have’… Sense of duty is not just about volunteering or sacrificing, but an environment that allows me to provide real care must also be present, so it would be nice to have guarantees for some of our rights as nurses.” (Participant #1)

## 4. Discussion

The present study was a qualitative study on the experience of new nurses in caring for COVID-19 patients in South Korea, which was analyzed using the phenomenological research method by Colaizzi [21]. Fourteen themes, divided into six theme clusters, and three categories were derived from the analysis of one-on-one interviews with the participants. While previous studies stated the experiences of experienced nurses, nurse managers, and nursing officers, the present study identified knowledge-related, technical, and psychological problems faced by recently graduated nurses in an isolated environment, without having much experience in performing clinical duties. Through such findings, the study also explored how they grew and built professional values, which can contribute to providing the foundation for developing strategies to help new nurses transition into qualified professional nurses.

The first category identified in the present study—“Fear as a new nurse who has not experienced infectious disease”—included the theme clusters “Difficulty from being inexperienced in new clinical duties” and “Fear of being a carrier of infection.” With respect to caring for patients with infectious disease, practical training is limited for students to protect them from the risk of infection, and consequently, they rarely gain appropriate experience [24]. The participants did not have such experience while in school, since practical training with hands-on experience was not included in the curriculum. Therefore, it is believed that new nurses faced even greater difficulties in accepting unfamiliar situations, such as an emerging infectious disease, while in the uncertain process of applying everything they had learned in college. Even experienced nurses had little experience with emerging infectious diseases, and thus, the categories of fear of infectious disease was similar [13,19], but the contents of theme clusters were different. New nurses were required to care for patients with emerging infectious diseases while lacking knowledge and skills; they were afraid because of their inexperience. Moreover, the participants struggled to make decisions in situations that required emergency care, but said care was being delayed due to the requirement of having to wear PPE. It is believed that it would be difficult to make ethical decisions when nurses have not been fully prepared by receiving knowledge on the ethical aspects of pandemics during their studies [24]. In the initial stage of the pandemic, the route of transmission was not clearly understood, and information was lacking. Accordingly, they were afraid of spreading the infection to their families and others around them. Moreover, although about half of the participants had received education regarding emerging respiratory infectious diseases during the MERS outbreaks, only 21.6% had received such education in a hospital [25]; education in hospitals and educational institutions was inadequate. There is a positive correlation between an increase in knowledge and attitude toward emerging infectious disease [25], and thus, there is a need for exposure to systematic educational programs starting from college.

The second category identified in the present study—“Physical and psychological burden in isolation environment”—included the theme clusters “Difficulties due to lack of professional skills” and “Burden due to playing too many roles as a nurse and the youngest member of the ward.” Wearing two pairs of gloves while having no confidence made it difficult to perform the IV injection, which is a delicate technique. Once nurses were inside an isolation unit, it was difficult to ask for assistance, and thus, they felt the pressure to resolve the situation on their own. Goggles, which are worn as PPE, can obscure their view due to fogging, and the weight of PPE limits movement when attempting to change the position of the patient. Therefore, the nurses first need to adapt to wearing PPE [20,26]. In particular, when new nurses make multiple mistakes in performing IV injections, they not only feel a sense of embarrassment, but also guilt because of harming the patient [27]. The isolated environment and wearing PPE made the situation even more difficult. Having to manage the batteries for electric respirators caused further difficulties in addition to the lack of skills. In cases where the participants’ hospital was designated a COVID-19 hospital, in providing care to patients with mild symptoms in the general ward the nurses only practiced simple skills, as compared with when they were caring for patients in the intensive care unit. Consequently, they gained extremely limited experience during a period when they should have been applying the various skills they learned in college, as a result of which they were worried about their work becoming stagnant. The lack of direct care competency among novice nurses with less than three years of experience is an important factor in turnover intention [28]. Accordingly, the findings of the study confirmed that the limited application of direct care during this period could be another source of stress for nurses.

Moreover, limiting the number of staff for patient care in order to prevent respiratory infectious disease transmission caused nurses to perform tasks that should otherwise be performed by guardians, caregivers, nurse assistants, or even cleaning staff, which caused them to feel overburdened with the work itself, as mentioned in a previous study [13]. This occurred due to more staff being needed in response to a significantly higher number of confirmed COVID-19 cases, which was different from the work burden due to lack of staffing caused by frequent isolation measures during patient care for MERS outbreaks [19]. Moreover, the participants faced tiring situations, being drenched in sweat as they exited the isolation unit after providing care while wearing PPE. However, they could not express the difficulties they faced as they were the youngest nurses. Therefore, there is a dire need to provide space and time for rest and emotional support to nurses who care for patients with infectious diseases. Moreover, the workforce needed to make all of this possible should be calculated [13], while institutional efforts at the government level should be made to reduce physical and mental fatigue among nurses under isolation.

The third category identified in the present study—“Building professional values”—included the theme clusters “Growing and learning with respect to knowledge, skills, and attitude” and “Recognized the social role of performing important work.” As new nurses, the participants thought about the roles they needed to play in the pandemic and tried to do their best to apply the knowledge they had learned and acquire new knowledge and skills as quickly as possible. Such findings reflected recognizing and putting in the efforts required to become nurses with professionalism, as they made an effort to acquire knowledge and skills to transition from new nurses to competent professionals while discovering how they were changing in a positive direction [29]. In early 2020, when the mass outbreak of COVID-19 in the Shincheonji religious facility in the Daegu area caused a specific religion to be the target of harsh criticism, nurses reminded themselves of the code of ethics for Korean nurses that stated that care should be provided to all patients without any discrimination; this reflected a process of building professional values. Moreover, they personally experienced how emotional care was needed while caring for patients experiencing delirium, which served as an opportunity to establish their attitude toward patient-centered nursing. The participants recognized their social role as professionals in the pandemic. In the midst of this, they were also aware of the issue of inadequate compensation that was raised during the pandemic while they were undergoing the process of building professional values. The issue of inadequate compensation is still being debated publicly, from the initial onset in 2020 to the present, in 2021 [8]. The government is planning to establish an infection control support funding system to provide institutional support, including planned payment of allowances to hospital-affiliated nurses [30]. Accordingly, people need to show interest for future institutional support policies to be effective. In addition, appropriate personnel support is also needed to ensure adequate rest for nurses.

The participants in the present study consisted of nurses from only three regions in South Korea (Seoul, Gangwon, and Daegu). Therefore, caution should be exercised when generalizing the findings. In this study, although there is no distinction between workers in the COVID-19-designated hospital and the COVID-19-designated ward itself, there is a difference in experience between the nurses in both places. Further in-depth research on nurses in COVID-19-designated hospitals is needed. 

Despite this, the significance of the present study can be found in the fact that it included new nurses from three different regions and that it identified the meaning behind the growth and development of professional values among new nurses despite the knowledge-related, technical, and psychological difficulties and burden they experienced in caring for patients with COVID-19.

## 5. Conclusions 

The present study explored the essence and meaning of the experience of caring for COVID-19 patients by new nurses in South Korea and used the data analysis method by Colazzi [21] for a comprehensive understanding of their experiences. The study results revealed that new nurses experienced fear of infection and felt burdened, in addition to being inexperienced in new clinical work that required caring for COVID-19 patients. They also experienced physical and psychological burdens in the isolated environment as the youngest and most inexperienced members. In the midst of this, they experienced the need to build professional values through their own efforts and growth.

Based on the findings of the present study, it is necessary to establish a national policy direction and provide institutional support to establish an appropriate compensation system for nurses who provide care for patients with infectious diseases in isolated environments. In preparation for other infectious diseases that may emerge in the future, senior colleges need to provide actual practical training before nursing students graduate, and the industry needs to establish strategies to provide regular education according to experience to ensure that new nurses transition into highly qualified professionals.

## Figures and Tables

**Table 1:** Themes, Theme clusters and Categories of the study.

Categories	Theme Clusters	Themes
Fear as a new nurse who has not experienced infectious disease	Difficulty from being inexperienced in new clinical duties	Did not learn or experience
		Feeling of lacking knowledge and decision-making skills
	Fear of being a carrier of infection	Worry about infecting family or close acquaintances
		Flustered by respiratory secretions despite wearing PPE
Physical and psychological burden in isolation environment	Difficulties due to lack of professional skills	Wearing PPE made difficulty due to lack of technical skills even worse
		Flustered when PPE was damaged
		Fear of forgetting nursing skills learned in school
	Burden due to playing too many roles as a nurse and the youngest member of the ward	All work is assigned to nurses
		Difficulties as the youngest member in the ward
Building professional values	Growing and learning with respect to knowledge, skills, and attitude	Applied knowledge for patient care/Continued observation and monitoring of objective values
		Continued practice and confirmation of PPE donning
		Need emotional care that is fair to all patients and appropriate for each situation
	Recognized the social role of performing important work	Positive public perception of nurses was a source of strength
		Inadequate compensation obscured by the sense of duty
